# Characterization of scanning orientation and lateral response artifact for EBT4 Gafchromic film

**DOI:** 10.1002/acm2.13992

**Published:** 2023-04-22

**Authors:** Hideharu Miura, Shuichi Ozawa, Toshiya Okazue, Tsubasa Enosaki, Yasushi Nagata

**Affiliations:** ^1^ Hiroshima High‐Precision Radiotherapy Cancer Center Hiroshima Japan; ^2^ Department of Radiation Oncology, Institute of Biomedical & Health Sciences Hiroshima University Hiroshima Japan

**Keywords:** film dosimetry, Gafchromic EBT4, lateral response artifact, scanning orientation effect

## Abstract

The purpose of this study was to investigate the impact of scanning orientation and lateral response artifact (LRA) effects on the dose‐response of EBT4 films and compare it with that of EBT3 films. Dose‐response curves for EBT3 and EBT4 films in red‐green‐blue (RGB) color channels in portrait orientation were created for unexposed films and for films exposed to doses ranging from 0 to 1 000 cGy. Portrait and landscape orientations of the EBT3 and EBT4 films were scanned to investigate the scanning orientation effect in the red channel. EBT3 and EBT4 films were irradiated to assess the LRA in the red channel using a field size of 15 × 15 cm^2^ and delivered doses of 200, 400, and 600 cGy. Films were scanned at the edge of the scanner bed, and the measured doses were compared with the treatment planning system (TPS) calculated doses at a position 100 mm lateral to the scanner center. At a dose of 200 cGy, the differences in optical density (OD) in the red, green, and blue color channels between EBT3 and EBT4 films were 0.035 (24.8%), 0.042 (49.7%), and 0.022 (64.4%), respectively. The EBT4 film slightly improved the scanning orientation compared to the EBT3 film. The OD difference in the different scanning orientations for the EBT3 and EBT4 films was 0.015 (6.8%) and 0.007 (3.9%), respectively, at a dose of 200 cGy. This is equivalent to a 20 or 10 cGy variation at a dose of 200 cGy. Compared with the TPS calculation, the measurement doses for EBT3 and EBT4 films irradiated at 200 cGy were approximately 16% and 13% higher, respectively, at the 100 mm off‐centered position. The EBT4 film showed an improvement concerning the impact of LRA compared with the EBT3 film. This study demonstrated that the response of EBT4 film to a dose in the blue channel was less sensitive and showed an improvement in the scanning orientation and LRA effects.

## 1 INTRODUCTION

Gafchromic films have been widely used in radiation therapy, such as patient‐specific quality assurance (QA) for proton therapy, intensity‐modulated radiotherapy (IMRT), volumetric modulated arc therapy (VMAT), stereotactic radiotherapy (SRT), and stereotactic body radiotherapy (SBRT).^1^, ^2^, ^3^, ^4^, ^5^, ^6^, ^7^, ^8^, ^9^, ^10^, ^11^, ^12^, ^13^, ^14^, ^15^, ^16^, ^17^, ^18^, ^19^, ^20^, ^21^, ^22^ Gafchromic EBT, EBT2, and EBT3 films were launched in 2004, 2009, and 2011, respectively. Currently, EBT3 film (Ashland Inc., Wayne, NJ, USA) is widely used for a dose range from 20 to 1 000 cGy. EBT‐XD film was released in the high‐dose range of 40−4 000 cGy for SRS and SBRT in 2015.^6^, ^21^, ^22^ In 2022, EBT4 film was released as a new generation of Gafchromic EBT film. The color of the EBT4 film is slightly darker yellow than that of the EBT3 film. According to the product specification notes, the configurations of EBT3 and EBT4 films are the same, as both have symmetric structures.^23^, ^24^ In addition to the configuration, the energy dependence, dose fractionation response, dose rate response, stability in light and dark, and uniformity are the same for both EBT3 and EBT4 films. EBT4 film could improve the signal‐to‐noise ratio with spatial resolution using the same chemical composition because the process of making the EBT4 film has changed from EBT3 film. A recent study on the new EBT4 film has reported high‐energy photon beams of nearly independent energy (6 MV, 6 MV Flattening‐Filter‐Free [FFF], 10 MV FFF, and 15 MV) were used in radiotherapy. However, for low‐energy photons (70 kV), there is an energy dependence, with a difference of up to 12%.^25^ Several studies have studied characteristics of the Gafchromic EBT film series and scanning procedures, including energy dependence,^4^, ^6^, ^22^ absorption spectra,^10^ film homogeneity,^11^ temperature dependence,^8^, ^12^ ambient light sensitivity,^12^ post‐exposure coloration,^4^, ^6^, ^12^ and variations among different lot numbers.^13^

EBT3 films exhibit two types of geometrical effects: scanning orientation^4^, ^6^, ^12^, ^16^, ^17^, ^20^ and lateral response artifact (LRA)^5^, ^7^, ^15^, ^16^, ^17^, ^18^, ^19^, ^20^, ^21^, ^22^ effects. The scanning orientation effect is the difference in the response of the scanner to the film orientation depending on the needle‐like microcrystal structure of the active layer of the film. This structure contributed to the polarization and anisotropic scattering of light by the film. The LRA is a non‐uniform response of the scanner at the position of the film perpendicular to the scan direction. The magnitude of the LRA depends on the irradiation dose and position of the film on the scanner bed.^5^, ^7^, ^15^, ^16^, ^17^, ^18^, ^19^, ^20^, ^21^, ^22^ The LRA should be considered for patient‐specific QA with large irradiation fields, such as head‐and‐neck and pelvic regions, due to incorrect measurement data at the off‐center position. We speculated that these two effects might be improved in the next‐generation EBT4 films due to the process of making EBT4 films.

We investigated the dose‐response curve, scanning orientation, and LRA effects for the EBT4 film and compared them with those of EBT3 films.

## 2 MATERIAL AND METHODS

### 2.1 Gafchromic EBT3 and EBT4 films

Gafchromic EBT3 (Lot #06152101) and EBT4 (Lot #07052202) films were used in this study. According to the product specification notes, the Gafchromic EBT3 and EBT4 films are composed of a 25 μm thick active layer by surrounded by two 125 μm thick matte polyester substrates. The active layer contains the active component, marker dye, stabilizers, and other components, giving the film a near‐energy‐independent response. The optimum dose range of EBT3 and EBT4 films is from 10 to 2 000 cGy and 2 to 1 000 cGy, respectively. Both the Gafchromic films were stored in a dark room, except during irradiation and scanning. The films were handled following the procedure described in the American Association of Physicists in Medicine (AAPM) Task Group 235 report.^26^

### 2.2 Dose‐response curve

A sheet of film (20.3 × 25.4 cm^2^) was cut into 4 × 4 cm^2^ pieces using a guillotine cutter. Each film was irradiated to create a dose‐response curve. One corner of each piece of the film was marked to track the orientation of the film. A piece of film was carefully placed on the central axis (CAX) in a water‐equivalent phantom (Kyoto Kagaku Co., Ltd, Kyoto, Japan) with 10 cm of buildup material above and below the film. The source‐to‐film distance was set at 100 cm. Twelve pieces of EBT3 and EBT4 films were used to create a dose‐response curve. One film piece was left unirradiated for the background. Irradiation was performed using a field size of 10 × 10 cm^2^ of a 6 MV beam produced by a Varian TrueBeam STx (Varian Medical Systems, Palo Alto, CA) linear accelerator. A total of 11 pieces were irradiated at the dose levels of 25, 50, 100, 150, 200, 300, 400, 500, 600, 800, and 1 000 cGy. Prior to any film irradiation, linac calibration was verified using a Farmer‐type ionization chamber (Model N30013; PTW, Freiburg, Germany) connected to a RAMTEC electrometer (Toyo Medic, Tokyo, Japan). Beam symmetry with a field size of 15 × 15 cm^2^ was confirmed using an IC PROFILER 2 (Sun Nuclear Corporation, Melbourne, FL).

### 2.3 Lateral response artifact

Both types of films (20.3 × 25.4 cm^2^) were cut into rectangular 20.3 × 4 cm^2^ pieces to investigate the LRA in portrait orientation. Each film was irradiated with a single dose of 200, 400, and 600 cGy in a water‐equivalent phantom with 10 cm of buildup material above and below the film at the center of a 15 × 15 cm^2^ open field using 6 MV photons.

### 2.4 Scanning protocol and analysis

Each irradiated film was scanned approximately 24 h after irradiation to minimize the effects of post‐irradiation coloration. An Epson Expression ES‐G11000 (Epson Seiko Corporation, Nagano, Japan) document scanner and Epson SCAN v3.49 software were used in this study. The scanners were turned on 30 min before scanning the images, and the warm‐up scans were repeated at least 10 times before scanning the films. The scanner was set to transmission mode with a 48‐bit red‐green‐blue (RGB) uncompressed tagged image file format (TIFF) image (16 bits per color channel), scan resolution of 75 dots per inch (dpi), and professional mode, with all available image correction methods, turned off. All the film pieces were positioned at the same location at the center of the scanning area. Each film used to create the dose‐response curve was scanned in both portrait and landscape orientations. Portrait and landscape orientations indicated that the long and short dimensions of the original film sheet were parallel to the scan direction, respectively. A paper ruler was used to assist in the accurate positioning of the films for scanning. A clear glass compression plate was placed above the films to mitigate natural film curling during the scans.^14^ Individual pixel values (PV) were extracted using IMAGEJ v1.49 software (National Institutes of Health, Bethesda, MD, USA). The median PV for each film for RGB color channels was measured on a region of interest (ROI) of 50 × 50 pixels to create a dose‐response curve. The edge of the film was carefully avoided when selecting the ROIs. PV was analyzed using a Microsoft Excel worksheet. Optical density (OD) was calculated using the following equation:^9^

$$\mathrm{OD}=OD_{exp}-OD_{unexp}=\log_{10}\frac{I_{unexp}}{I_{exp}}$$

where *I _unexp_* and *I_exp_* are the PV readings for the unexposed and exposed film pieces, respectively.

To emphasize the LRA, the films exposed to the 15 × 15 cm^2^ open field irradiation were scanned in portrait orientation at the positions laterally shifted by approximately 5 cm, and one edge of the film touched the edge of the scan window. Measurement dose on each film was compared with the calculated dose by the treatment planning system (TPS) in the absolute mode. Owing to its high sensitivity, the red channel was investigated for LRA in scanning orientation. Although the magnitude of the LRA differs quantitatively in portrait and landscape orientations, we chose the portrait orientation to investigate the LRA because patient‐specific QA films for conventional IMRT and VMAT plans with large fields are scanned considering the scanner size.

## 3 RESULTS

**FIGURE 1:**
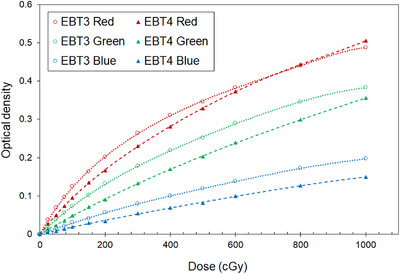
Dose‐response curves as a function of irradiated dose for EBT3 and EBT4 films in the red, green, and blue color channels.

Figure 1 shows the dose‐response curves in three color channels for the 0−1 000 cGy dose range in the portrait orientation. For both types of films, the red‐colored channel was the most sensitive to the irradiated dose. The dose‐response curve in the red channel of the EBT 3 film showed a high gradient up to doses of 800 cGy. For the green and blue channels, the OD was lower for EBT4 than for EBT3 films at doses ranging from 0 to 1 000 cGy. At a dose of 200 cGy, the differences in OD in the red, green, and blue color channels between the EBT3 and EBT4 films were 0.035 (24.8%), 0.042 (49.7%), and 0.022 (64.4%), respectively. At a dose of 1 000 cGy, the differences in OD in the red, green, and blue color channels between EBT3 and EBT4 films were 0.018 (0.2%), 0.027 (13.5%), and 0.048 (37.5%), respectively. The dose‐response of the EBT4 film in the blue channel was significantly less sensitive than that of the EBT3 film.

**FIGURE 2:**
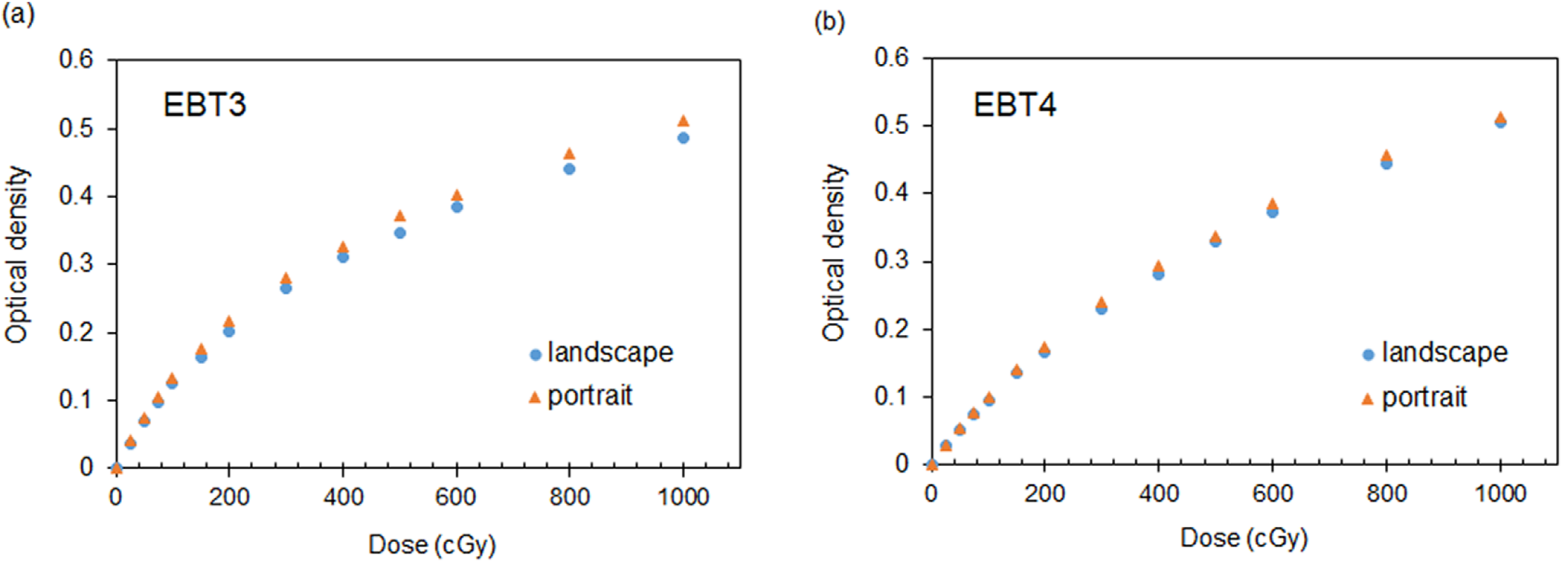
Dose‐response curves in the red color channel as a function of irradiated dose for (a) EBT3 and (b) EBT4 films in landscape and portrait orientation.

Figure 2 shows the dose‐response curves in the red channel in the landscape and portrait orientation for the EBT3 and EBT4 films. The OD in the portrait orientation was always higher than in the landscape orientation. The OD differences in the different scanning orientations for EBT3 and EBT4 films were 0.004 versus 0.001, 0.015 versus 0.007, and 0.024 versus 0.007 at doses of 25, 200, and 1 000 cGy, respectively. All irradiation doses have large OD differences in scanning orientation for EBT3 and EBT4 films, such as 0.015 (6.8%) and 0.007 (3.9%) OD changes at a dose of 200 cGy. This is equivalent to a 20 or 10 cGy variation at a dose of 200 cGy.

**FIGURE 3:**
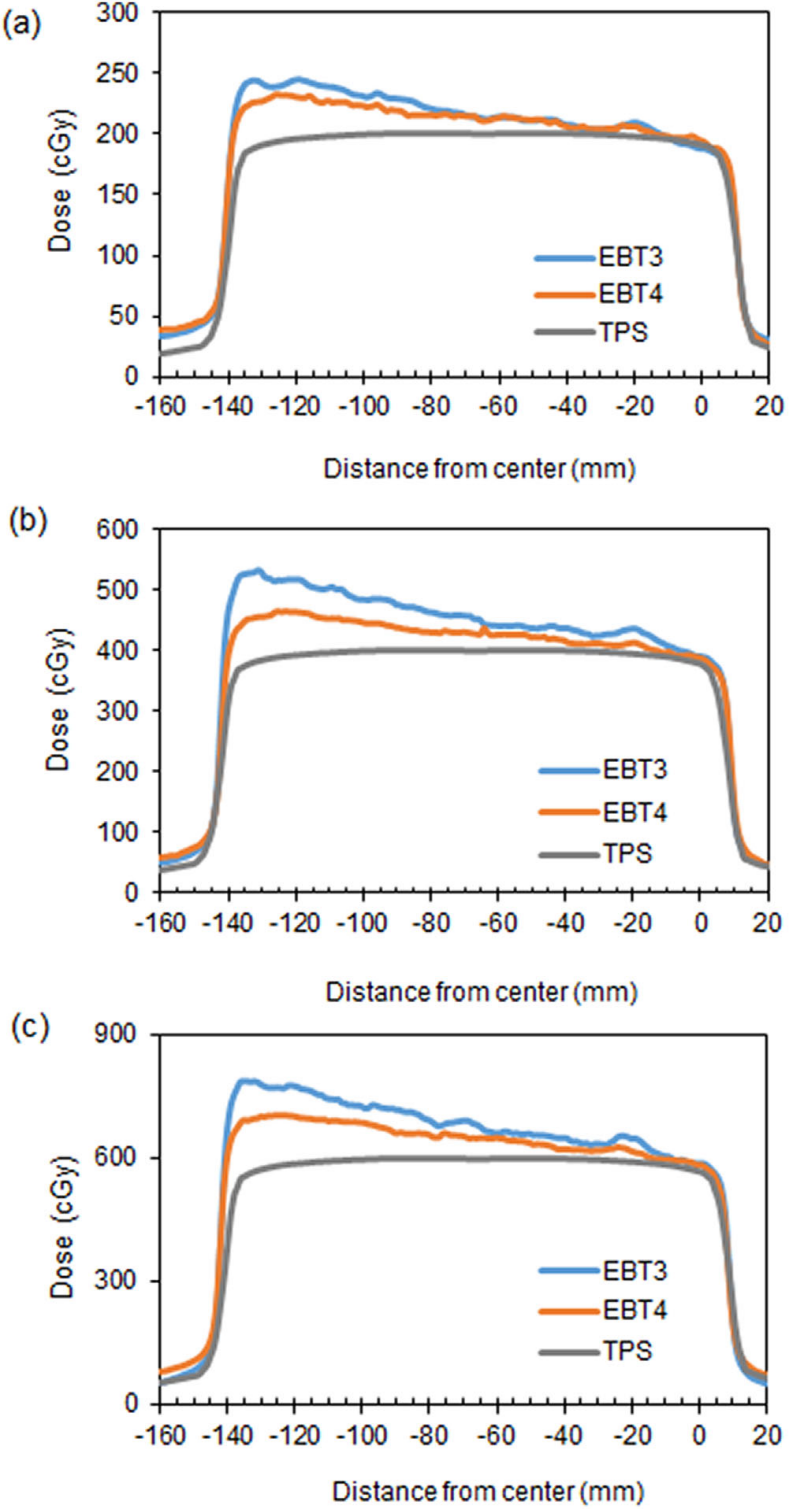
Lateral dose profiles acquired using EBT3 and EBT4 films in red color channel irradiated with (a) 200, (b) 400, and (c) 600 cGy doses, with a field size of 15 × 15 cm^2^ open field. The measured profiles were acquired perpendicular to the scan direction. The absolute dose was calculated with a treatment planning system (TPS) to compare with film measurement. The 0 mm position corresponds to the center of the scanner.

Figure 3 shows transverse dose profiles in the lateral direction of EBT3 and EBT4 films irradiated with a field size of 15 × 15 cm^2^ open field and scanned at one of the extreme lateral position on the scanner bed. Dose‐response of the EBT3 and EBT4 films at the off‐center position is high with respect to the center of the scanner. The LRA for both types of films depended not only on the position of the film position but also on the irradiated dose. The measurement doses for EBT3 and EBT4 films irradiated with the 200, 400, and 600 cGy were approximately 16.4% versus 12.5%, 21.0% versus 11.7%, and 21.2% versus 14.8% higher than TPS calculation at the off‐center positions of 100 mm, respectively. EBT4 film exhibited an improvement for LRA compared to EBT3 film, but the need for LRA correction in large fields is not eliminated by using EBT4.

## 4 DISCUSSION

We investigated the EBT4 film for dose‐response, scanning orientation, and LRA effects and compared the results with those of the EBT3 film. Compared with the ODs of the EBT3 film, the EBT4 showed that ODs in the red, green, and blue channels tended to be lower, with a decrease of 24.8%, 49.7%, and 64.4% at a dose of 200 cGy, respectively. Our measurements showed that the dose‐response of the EBT4 film in the blue channel showed the least sensitivity among the three channels. The yellow marker dyed absorbs most of the blue and ultraviolet wavelengths and makes a relatively small contribution to the OD in the blue color channel. The dark‐yellow color in the EBT4 film likely absorbed more light in the blue region of the visible spectrum than the light‐yellow color in the EBT3 film.

Improving the scanning orientation and LRA effects could be related to the process improvement of the active fluid in EBT4. This information was obtained by personal communication with the vendor. Differences in response with respect to the scan direction are well‐known and are affected by the size and shape of the crystals of the active component.^4^, ^6^, ^16^ Our measurement showed that the greatest difference in the dose‐response due to scanning orientation between EBT3 and EBT4 films in the red color channel was approximately 9.5% and 4.8% at a dose of 25 cGy, respectively. At a dose of 200 cGy, the differences between EBT3 and EBT4 due to scanning orientation were 6.8% and 3.9%, respectively. As the dose delivered to the film increased, the difference in the OD decreased. In practice, the EBT4 film also should be scanned in the same direction at the time of dose response curve and patient specific QA to reduce the potential systematic error.

OD or PV measured using flatbed scanners show variations depends on the position of the scanner.^16^, ^17^ Many previous studies have reported that the LRA influences the optical path length of the film at an off‐center position, and the interaction of the polarization and scattering of light leaving the film and the mirror system guiding the light to the charge‐coupled device (CCD) detectors.^16^, ^17^ The measured data scanned at one of the extreme lateral positions on the scanner bed showed prominent characteristics of the dose profiles at the off‐center position. In our study, the LRA of the EBT4 film was slightly lower than that of the EBT3 film at all irradiation doses. The EBT4 film showed improvement against the LRA by 3%, 8%, and 7.5% at doses of 200, 400, and 600 cGy, respectively, at a position 100 mm off‐center on the scanner, compared with the EBT3 film. Although direct comparisons among several studies are difficult because of the differences in irradiation dose and evaluation distance from the center, the type of film, and scanners, most researchers concluded that the magnitude of LRA depends on the dose and the lateral distance from the center of the scanning bed. In our study, the OD of the EBT4 film in the red channel was lower than that of the EBT3 film. Several methods have been proposed to correct the LRA of flatbed scanners in radiochromic film dosimetry.^7^, ^15^, ^18^, ^19^ A combination of EBT4 film and LRA correction will provide a more robust film dosimetry for treatment plan QA with a large irradiation field. After the study, the authors noticed that LRA still existed for EBT4. LRA correction methods should still be applicable to EBT4 film.

A limitation of this study is that only a single lot of EBT3 and EBT4 films were investigated for comparison. Both types of dose‐response curves might be different for different lot numbers.^13^ Another limitation was that only a single red channel was investigated for the scanning orientation and LRA effects. The LRA effect in the green and blue color channels is much less than that in the red color channel.^18^, ^19^, ^21^

## 5 CONCLUSIONS

This study investigated the dose‐response, scanning orientation, and LRA for EBT3 and EBT4 films. Our results demonstrated that the dose‐response of the EBT4 film in the blue channel was less sensitive and the EBT4 film exhibited reduced scanning orientation and LRA effects compared with the EBT3 film. The process of making the EBT4 film contributed to the improvement of the scanning orientation and LRA effects. Despite the improvement in scanning orientation and LRA for EBT4 over EBT3 films at all dose ranges, scanning orientation and LRA were non‐negligible. LRA is a fundamental feature of flatbed scanners and should be corrected for the response at the off‐center position of the scanner bed.

## AUTHOR CONTRIBUTIONS

All authors contributed substantially to the design of the study. Hideharu Miura conducted the study and all authors contributed to the analysis and interpretation of the results. Hideharu Miura drafted the manuscript and all authors provided critical feedback on revising the manuscript for important intellectual content and all authors gave their final approval of the version to be published.

## CONFLICT OF INTEREST STATEMENT

The authors declare no conflicts of interest.
